# Fiber-Optical Sensors: Basics and Applications in Multiphase Reactors

**DOI:** 10.3390/s120912519

**Published:** 2012-09-13

**Authors:** Xiangyang Li, Chao Yang, Shifang Yang, Guozheng Li

**Affiliations:** Key Laboratory of Green Process and Engineering, Institute of Process Engineering, Chinese Academy of Sciences, Beijing 100190, China; E-Mails: xyli@home.ipe.ac.cn (X.L.); yangshi.fang@163.com (S.Y.); gzli@home.ipe.ac.cn (G.L.)

**Keywords:** fiber-optical sensor, probe, multiphase reactor, local flow characteristics

## Abstract

This work presents a brief introduction on the basics of fiber-optical sensors and an overview focused on the applications to measurements in multiphase reactors. The most commonly principle utilized is laser back scattering, which is also the foundation for almost all current probes used in multiphase reactors. The fiber-optical probe techniques in two-phase reactors are more developed than those in three-phase reactors. There are many studies on the measurement of gas holdup using fiber-optical probes in three-phase fluidized beds, but negative interference of particles on probe function was less studied. The interactions between solids and probe tips were less studied because glass beads *etc.* were always used as the solid phase. The vision probes may be the most promising for simultaneous measurements of gas dispersion and solids suspension in three-phase reactors. Thus, the following techniques of the fiber-optical probes in multiphase reactors should be developed further: (1) online measuring techniques under nearly industrial operating conditions; (2) corresponding signal data processing techniques; (3) joint application with other measuring techniques.

## 1. Introduction

Multiphase reactors are the most important equipment in the chemical industry, where chemical reactions take place involving several reactants in different phases. To describe and design multiphase reactors, traditional approaches based on empirical rules and correlations rely to a large extent on the measurements made under conditions as relevant as possible to industrial practice. Modern computational fluid dynamics (CFD), which has been extensively used for the numerical simulation of multiphase reactors [1–5], also requires the information on local and transient flow characteristics to build precise physical models. Reliable measuring techniques are therefore needed for the rational description and design of multiphase reactors.

The measurement techniques for multiphase reactors can be classified as invasive (such as fiber-optical probes [6,7], impedance probes [8,9], heat transfer probes [10,11] and ultrasound probes [12,13]) and non-invasive techniques (including optical techniques [14–18] and tomography [19–23]). Boyer *et al.* [24] have reviewed and compared them in detail. Invasive measuring techniques cannot be avoided though non-invasive techniques are intensively developed for the analysis of multiphase flows. This is particularly true for highly turbulent systems, due to two main reasons: (i) in case of nearly industrial operating conditions (particular physico-chemical environment, opaque walls, high gas holdups or solid concentrations, *etc.*), non-invasive techniques become ineffective; (ii) non-invasive techniques are often difficult and expensive for industrial applications.

In all of non-invasive techniques, fiber-optical probes may be the most promising ones because of their inherent advantages such as harsh environment tolerance and very small size, which will be discussed in Section 2.1. Benefitting from great developments in the optoelectronic and fiber-optical communications industries, great progress has also been made in fiber-optical sensor technology with vastly improved optical and mechanical properties and lower cost of the components over the past 30 years. As a result, the ability of fiber-optical sensors to displace traditional sensors for rotating, accelerating, electric and magnetic field measurements, temperature, pressure, acoustics, vibration, linear and angular positions, strain, humidity, viscosity, chemical measurements, and a host of other sensor applications has been enhanced [25]. A number of useful reviews such as those by Kersey [26], Grattan and Sun [27] and Lee [28], and monographs such as those by Yin *et al.* [29] and Udd *et al.* [30] have been produced over the years. Progresses in fiber-optical sensor technique open a door for the measurements of multiphase reactors and can offer many important measurement opportunities and great potential applications in this area.

The aim of this paper was to review the most significant developments and applications of fiber-optical probes for multiphase reactors. The remainder of this paper is organized as follows: in the next section, the basics of fiber-optical sensors are presented. Then, significant developments and applications of fiber-optical sensors/probes for multiphase reactors (involving gas-solid, liquid-solid, gas-liquid, liquid-liquid, gas-liquid-solid systems) will be introduced. Finally, the future research trends in the field of fiber-optical sensors/probes for multiphase reactors will be discussed and summarized.

## 2. Fiber-Optical Sensor Basics

### 2.1. Why Fiber-Optical Sensors?

The inherent advantages of fiber-optical sensors range from their: (1) harsh environment capability to strong EMI (electromagnetic interference immunity), high temperature, chemical corrosion, high pressure and high voltage; (2) very small size, passive and low power; (3) excellent performance such as high sensitivity and wide bandwidth; (4) long distance operation; and (5) multiplexed or distributed measurements, were heavily utilised to offset their major disadvantages of high cost and end-user unfamiliarity [29].

### 2.2. Compositions of Fiber-Optical Sensors

**Figure 1.**
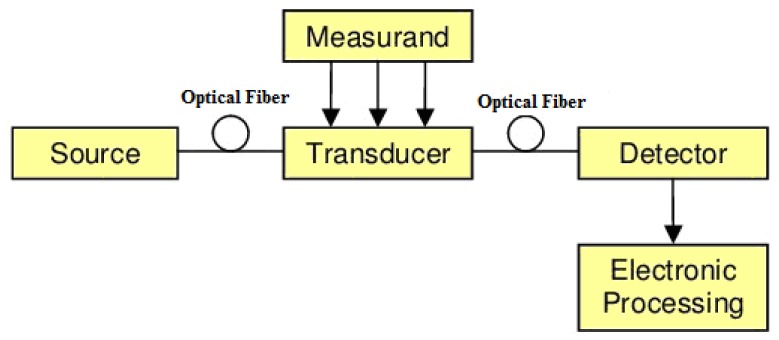
Basic components of a fiber-optical sensor system [25].

As shown in Figure 1, a fiber-optical sensor system consists of an optical source (laser, LED, laser diode, *etc.*), optical fiber, sensing or modulator element transducing the measurand to an optical signal, an optical detector and processing electronics (oscilloscope, optical spectrum analyzer, *etc.*) [25]. The advent of laser opens up a new world to researchers in optics. Light sources used to support fiber-optical sensors produce light that is often dominated by either spontaneous or stimulated emission. A combination of both types of emission is also used for certain classes of fiber-optical sensors.

### 2.3. Fiber-Optical Sensor Classifications

Fiber-optical sensors are often loosely grouped into two basic classes referred to intrinsic, or all-fiber and extrinsic, or hybrid sensors. The intrinsic fiber-optical sensor has a sensing region within the fiber and light never goes out of the fiber. In extrinsic sensors, light has to leave the fiber and reach the sensing region outside, and then comes back to the fiber [29]. Furthermore, fiber-optical sensors can also be classified under three categories [25]: the sensing location, the operating principle and the application, as seen in Table 1.

### 2.4. Current Applications

Fiber-optical sensors have been the topic of considerable amounts of research for the past 30 years and their application fields are being extended continuously in two major fields, *i.e.*, as a direct replacement for existing sensors and the development/deployment of fiber-optical sensors in new areas. To date, the most highlighted application fields of fiber-optical sensors are in large composite and concrete structures, electrical power industry, medicine, chemical sensing, and gas and oil industry. A wide range of environmental parameters such as position, vibration, strain, temperature, humidity, viscosity, chemicals, pressure, current, electric field and several other environmental factors have been widely monitored. More detailed information on the applications can resort to references [25] and [29].

## 3. Application of Fiber-Optical Probes in Multiphase Reactors

A multiphase system with gas as the dispersed phase may be a gas-liquid or gas-liquid-liquid or gas-liquid-solid system. Due to the too small differences in refractive index between gases and organic liquids, fiber-optical probes are rarely utilized in experimental studies on the measurement of gas-phase characteristics in a gas-liquid-liquid system. So the discussion on this system is combined with the gas-liquid one. The gas-liquid-solid system has two dispersed phases and the complicated effects between different phases make the experimental studies more difficult. So in Section 3.1, the main concern is the measurements in gas-liquid reactors, and the studies on gas-liquid-solid reactors are involved in Section 3.4. In Sections 3.2 and 3.3, the experimental studies on the measurements of the drop-phase and solid-phase characteristics are dealt with, respectively.

### 3.1. Measurements of Gas-Phase Characteristics

In gas-liquid reactors, more information about the local gas-phase characteristics such as bubble-size distribution, bubble velocity, local gas holdup and bubbling frequency is useful for monitoring the homogenization of aeration in the whole volume of the reactor and for predicting the mass-transfer characteristics between gas and liquid. Needle probes are always used. Single-tip probes lead to gas fraction and double-tip probes allow measurements of bubble velocity, time-averaged local interfacial area and mean bubble chord length. As a needle probe, an infra-red light beam is conducted along the fibre to the needle tip, where the thin fibre ends usually as a sharp cone so as to pierce small bubbles. Following optic laws, this tip transmits the light beam away when submerged in liquid, or reflects it back to the electronic receiver when it is surrounded by gas. An optoelectronic device (phototransistor) delivers an analog output signal in proportion to the received light intensity [24].

The techniques using fiber-optical probes in this area are more developed. Over the past decades, there have been a number of designs developed for the fiber-optical probes in measurement of gas-liquid flows, such as mono-fiber probes, double-tip optical probes, U-shaped probes and prism-linked probes. Boyer *et al.* [24] have reviewed the fiber-optical probes in measurement of gas-liquid flows in detail. So the focus of this paper is put only on the new development hereafter.

#### 3.1.1. Measurement in a Multi-Dimensional Flow

The interfacial area concentration (IAC) is defined as the interfacial area existing in a unit volume of the mixture and specifies the geometric capability of interfacial transfer. The principle of IAC measurement with a double- or four-sensor probe was originally proposed by Kataoka *et al.* [31]. Then, the double-sensor probe method was improved by Hibiki *et al.* [32], which can measure the IAC effectively merely in a one-dimensional flow because of its two main assumptions: (1) the interfacial velocity can be approximated by using the ratio of the separation of two sensor tips and the time difference when the interface passing the two sensor tips; and (2) the bubble is spherical in shape.

The bottleneck problem is how to deal with receding interfaces, namely the interfaces that touch the rear sensor tip(s) ahead of the front sensor tip, in a multi-dimensional two-phase flow measurement. Shen *et al.* [33] derived the interfacial measurement theorem relating the local instantaneous interfacial velocity to local measurable velocities based on the vector triangle analysis and improved the four-sensor probe method. Using the improved four-sensor probe method, not only oncoming interfaces (namely the interfaces that touch the front sensor tip ahead of all rear sensor tips) but also receding ones could be measurable. Then, the study on the error reduction, evaluation and correction for the intrusive optical four-sensor probe measurement in multi-dimensional two-phase flow was conducted by Shen *et al.* [34]. With regard to the unsatisfactory measurement errors for the receding (respectively 31% and 38% in IAC and void fraction) and transversal bubbles (up to 30% for the maximum underestimation of IAC), a correction method for the four-sensor probe measurement in a multi-dimensional two-phase flow was proposed. More measurements have to be conducted to validate the effectiveness of this correction method hereafter.

**Figure 2.**
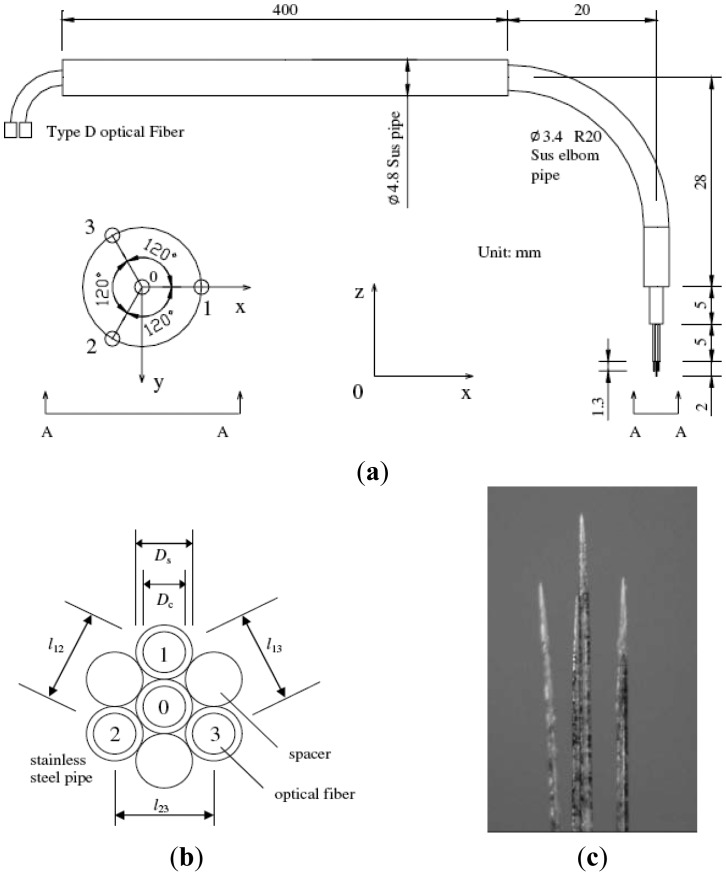
Design of typical optical four-sensor probes [34]: (**a**) full view of a four-sensor probe; (**b**) a hexagon module; (**c**) photo.

The typical optical four-sensor probe is designed as shown in Figure 2 by using a fiber with 125 μm in clad diameter (*D*_c_) and 50 μm in core diameter (*D*_0_). The supporting stainless steel pipes, with 0.35 mm in outer diameter (*D*_s_), 0.09 mm in thickness and 40 mm in length, were arranged in a hexagon in a module as shown in Figure 2(b). The four-sensor probe was made by threading the fibers with required tip shapes through the supporting pipes. An example of a fabricated conical optical four-sensor probe is illustrated in Figure 2(c).

#### 3.1.2. Applications in Complex and Chaotic Flows

Under relevant industrial operating conditions (such as high gas and liquid flow rates, large bubbles and vortices), the flows in multiphase reactors are complex and chaotic. It is therefore difficult to perform reliable experimental measurements to obtain the local data, especially bubble size values, but the fiber-optic probes can provide local gas hold-up and bubbling frequency directly even if the sensor is not strictly flow oriented. With a specific signal treatment and under some assumptions such as exclusively vertical bubble motion, isotropy of turbulence and regular bubble shapes (spherical or ellipsoidal), bubble velocity and bubble size may also be derived [35–37]. Chaumat *et al.* [38] pointed out that bubble size distribution was very difficult to obtain by adopting this kind of treatment for chains of distorted tumbling bubbles. In a subsequent study, Chaumat *et al.* [39] found that the optic probe provided the most probable ascendant velocity based on the comparison between the measured most probable velocity and the actual gas velocity. Thus, a methodology for double optic probe data treatment was established for more complex flows (high bubble density, liquid and bubble loops). In this methodology, the velocity was estimated through the most probable velocity issued from the intercorrelation of both raw signals and the equation for the average bubble estimation. The most probable bubble velocity was also used to substitute the bubble velocity. Even if the obtained data are not very precise yet, some efforts are still necessary in this way.

**Figure 3.**
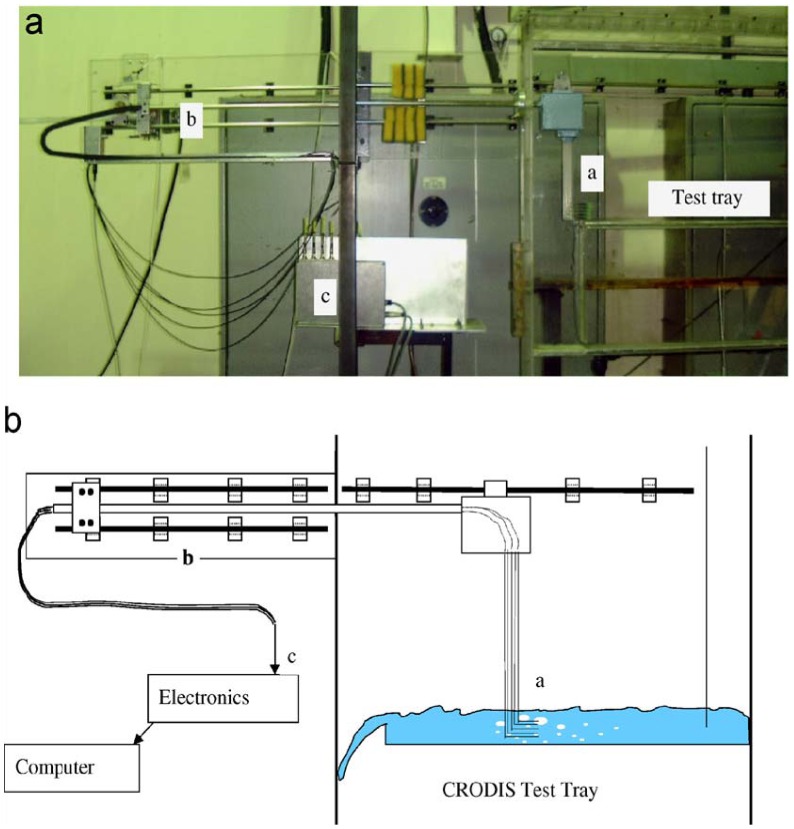
(**a**) Photograph of a flying optical probe system; (**b**) Diagram of a flying optical probe system [42].

In the most common approach, the probe was kept stationary at a fixed location in the flow field and the probe tip was oriented opposite to the main stream flow. However, in some multiphase equipment such as sieve tray distillation columns, the chaotic and multi-directional nature of the flow above a sieve tray made it almost impossible to find such an orientation. Calderbank and Pereira [40] attempted to address this difficulty by using a compound probe consisting of four sensors, and only the signals coming from the bubbles rising vertically and coaxially with all four sensors were considered to be valid. These bubbles, however, may not completely represent the whole spectrum of bubble sizes which exist in the multi-dimensional flow system. Also, the bubble count rate gleaned effectively from the compound probe was quite low, e.g., 200 bubbles every 3 h as reported by Raper *et al.* [41]. To tackle the measurement problem encountered in such highly chaotic and turbulent systems, the “flying optical probe” was successfully developed by Hu *et al.* [42]. As shown in Figure 3, an array of optical probes was driven (“flown”) across a simulated distillation tray at a fast but constant speed. The bubble layer on the tray then appeared in a framework moving with the probe as a bubble flow moving towards the probes and the probe responses could be analyzed to give BSDs (bubble size distributions) on the tray.

#### 3.1.3. Operation in Organic Liquids

**Figure 4.**
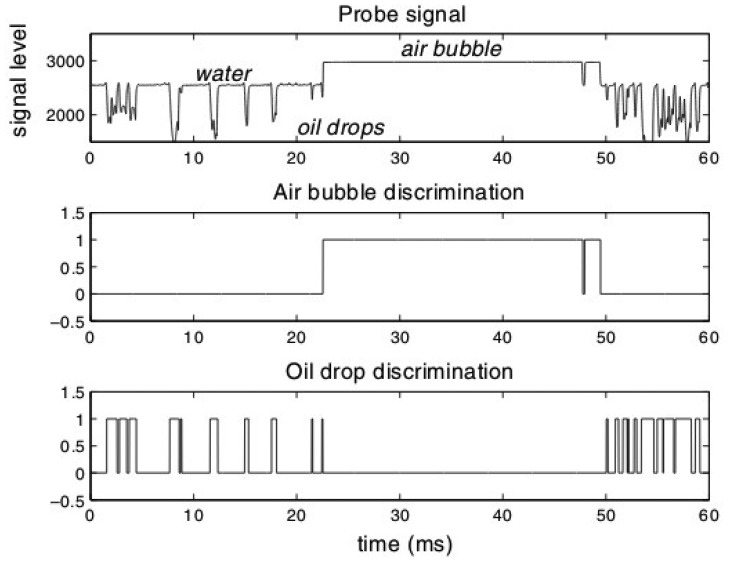
Probe detection and data processing for air bubbles in oil-in-water flow [43].

One of the drawbacks of fiber-optical probes as pointed out by Boyer *et al.* [24] is that they do not operate in all organic liquids because of the too small differences in refractive index with the gas phase. On the other hand, organic liquids often appear in gas-liquid chemical reactors. Descamps *et al.* [43] thought that the fiber-optical probe can be discriminated between oil, air and water in theory, even if the difference in refractive index between them is small. In order to minimize the effects brought by organic liquids, they used an algorithm developed by Harteveld [44] to perform a careful experimental data processing. A data processing example for air bubbles in oil-in-water flow is shown in Figure 4. The calculation of the time-averaged void fraction has been validated and proved to be accurate for gas-liquid flow (error less than 5%) [44,45]. Concerning bubble size and velocity, some comparisons have been made between fiber-optical probe measurements and high speed video recordings. After processing, the value given by the optic probe exhibits an uncertainty range of 15%. Though it is relatively high, it is still a valuable attempt.

#### 3.1.4. A Novel Method to Measure Mixing Quality

**Figure 5.**
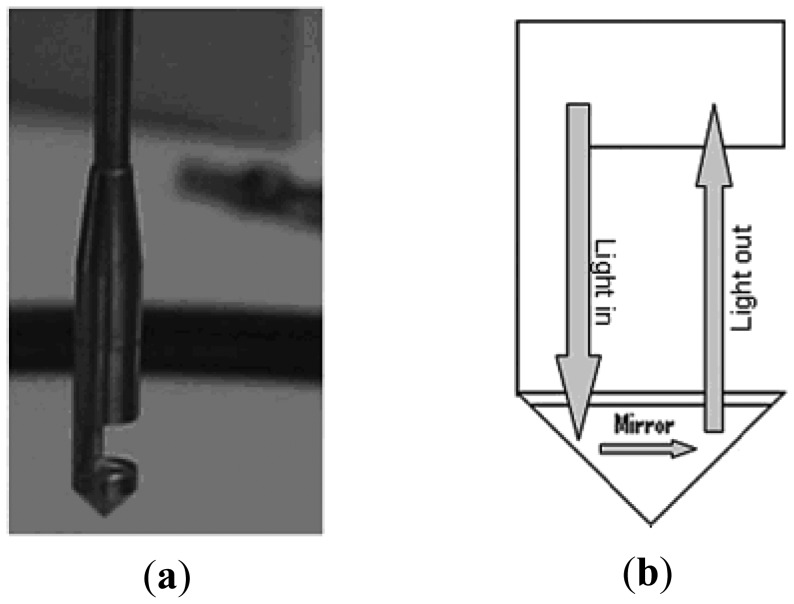
Fiber-optical immersion probes with replaceable tips: (**a**) image of one probe; (**b**) schematic drawing of the UV-VIS light path [52].

Another development worth mention is a novel method using fiber-optical probes to measure the quality of mixing in multiphase reactors. As is known, the mixing time is an important parameter used to characterize the quality of mixing [46]. In order to define mixing time, the “95% criterion” is often chosen [47–50]. The techniques most commonly used to determine mixing time are conductivity and colorimetric methods [51]. The conductivity technique cannot be applied where the rheological properties are affected by the presence of salts and is bothered with the interference of the existence of dispersed phases. Mixing time cannot be measured using these techniques when the actual chemical process of interest is operative, either. Thus, a UV/Visible (UV-VIS) *in situ* fiber-optical probe coupled with a McPherson spectrograph was designed to collect the spectra of non-reactive tracers at a maximum acquisition rate of 100 spectra s^−1^ using a full vertical binning acquisition mode and 41.7 spectra s^−1^ when using a multitrack acquisition mode [52]. This fiber-optical immersion probe is shown in Figure 5, where the light from a tungsten light lamp passed through a focusing lens and the sample solution filling a gap of 5 mm.

Then it was reflected back by a mirror placed at the base of the replaceable tip. The light traveled double the 10 mm gap between the focusing lens and the mirror. The spectra acquired were processed using a Savitzky-Golay smoothing filter algorithm and analyzed according to the method proposed by Ruszkowski [53], to give mixing time values. Using this technique, mixing time was measured in three stirred vessels and the results agreed well with those obtained from a conductivity technique and with a correlation proposed in the literature [54]. This technique also suits for monitoring other fast reactions and concentration differences in reactors.

### 3.2. Measurements of Solid-Phase Characteristics

**Figure 6.**
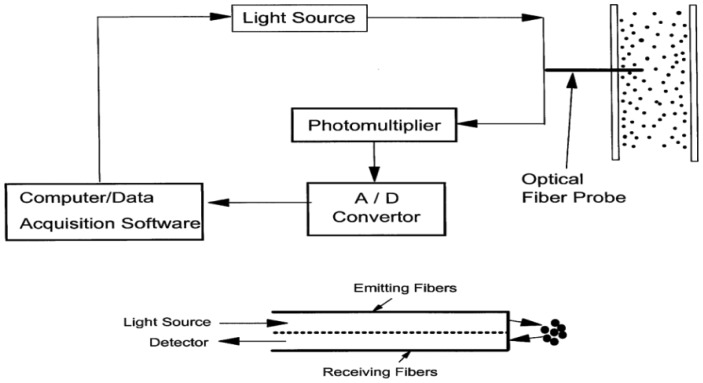
The fiber-optical probe system for solids holdup measurement.

The solids concentration, the particle velocity and the corresponding solids flux are considered as the main parameters of characterizing gas-solid and solid-liquid two-phase flow structures. The local solids holdup is measured using the optical fiber solids concentration probes and the local particle velocity is measured by the optical fiber particle velocity probe. The principles also rely on the difference in refractive index of the probe and the surrounding media. A classic optical fiber solids concentration probe made by our group (shown in Figure 6) has a 3.8 mm o.d. stainless steel probe tip, containing approximately 8,000 emitting and receiving quartz fibers, each 15 ìm in diameter. These fibers are arranged in an array consisting of alternate layers of emitting and receiving fibers, within a 1.5 mm square area at the center of the probe tip. A bundle of fiber projects light onto the passing cluster of particles. The other interspersed fibres in the bundle act as light receivers transmitting the light reflected by the particles to a photo transistor which converts the light into an electrical signal. An amplifier increases the resulting signal to a voltage range 0–5 V and then to an A/D converter. The relative error of the probe measurement is 1/256 of the full range for *ε*_s_ measurements. The particle velocity probe uses one or more fibers (or fiber bundles) to project light (e.g., laser) on the flow and two or more fibers (or bundles) to detect the reflected light.

#### 3.2.1. Gas-Solid System

In gas-solid systems, most studies on solids distribution were conducted by measuring the axial profiles of pressure gradient. Although this method is reliable and easy to apply, it is incapable of obtaining local solids concentration and other quantities. As is known, non-invasive techniques have their limitations and become ineffective in case of nearly industrial operating conditions (particular physico-chemical environment, opaque walls, solid concentrations, *etc.*) [24]. Reflective-type fiber-optical probes, a type of intrusive techniques, overcome these drawbacks and have been shown to be an effective tool in measuring local solids holdup and particle velocity in the riser by a number of researchers [55–60]. These probes, whose tips may be either single- or multi-fiber type, emit light to illuminate a small volume of particles passing by the probe tips and detect the reflected light intensity with electrical impulse output. They are good for local properties, effective under a wide range of conditions, and applicable for most powder types in both gas and liquid media measurements. Additionally, they are nearly free from interference of extreme temperature, humidity, electrostatic and electromagnetic fields, *etc.* [59]. Razzak *et al.* [61,62] developed an integrated system of ERT and optical fiber probes to measure the local radial distribution holdups in a GLSCFB riser.

The accuracy of solids volume concentration measurements depends strongly on the calibrating method used for the fiber-optical probe, because a calibration relationship between the output signal of the fiber-optical probe (*i.e.*, readings of the digital integrator) and solids volume concentration is needed before measurement. Earlier works on fiber-optical probe calibration were conducted based on linear or near-linear assumptions and a calibration was established by comparing the signals to two concentration values obtained by other techniques such as γ-ray [63–65] and static pressure drop measurements [66,67]. These techniques were not accurate and a linear relationship was hardly justified [59]. Then, a non-linear model was developed for a single fiber reflection probe [68–70]. A calibrating curve was obtained in a water-solid fluidized bed and then used it in a gas-solid system. Another technique reported by Matsuno *et al.* [71] employed the free-falling particles at their terminal velocities in a gas-solid system for calibration. However, the application of this method is limited because the calibration was in low concentration. Though Herbert *et al.* [72] greatly improved past calibration techniques, there are still some limitations. In the study of Zhang *et al.* [59], a multi-fiber optical reflection probe was uniquely calibrated in a downer to obtain quantitatively precise solids holdup. An iterative procedure was utilized to modify the initial calibration curves, which was verified both theoretically and practically.

#### 3.2.2. Solid-Liquid System

Also, solid concentration as a main parameter was of major concern in solid-liquid systems. The measurement in such a system is relatively easy, so most of the measuring techniques had successful applications and reported in the literature. Warsito and Fan [73] used an electrical capacitance tomography (ECT) to distinguish the three phases in a gas-liquid-solid fluidized bed qualitatively. The radial non-uniformity in a LSCFB was first reported using a conductivity probe [74] and a fiber-optical probe [75]. The measurement of the local solids concentration in a stirred tank with an elliptical bottom, using a PC-6A fiber-optical probe, was conducted in an obscured environment to prevent daylight from interfering with the optical technique by Shan *et al.* [76]. The procedure for fiber-optical calibration in liquid-solid systems is the same as that in gas-solid systems.

### 3.3. Measurements of Drop Characteristics

Liquid-liquid dispersions in stirred vessels or mixers are often encountered in the chemical, pharmaceutical, metallurgy and food industries. For designing, controlling and optimizing these systems, exact knowledge about drop size distribution (DSD) and its transient behavior when energy input, temperature or composition subjected to certain changes is of major importance. For several decades, some authors have tried to use the population balance equation (PBE) to predict the evolution of the DSD. However, the key challenge associated with the implementation of predictive PBE models is the experimental determination of drop breakage and coalescence functions which represent two main classes of mechanisms involved during emulsification. As highlighted by Sathyagal *et al.* [77] and O'Rourke and MacLoughlin [78], it required reliable measurements of transient or evolving size distributions over extended periods in contacting configuration of practical interest.

Thorough reviews of the drop sizing methods used were given by O'Rourke and MacLoughlin [78] and Brown *et al.* [79]. In various sampling methods, the samples were withdrawn over time from the vessels, which were later diluted or stabilized, prior to their measurements [80–84]. These sampling techniques neither guaranteed that the drop sizes were frozen, nor that they were preserved during the sampling [85,86]. So the transient drop behavior was not obtained with confidence. The online measurement techniques should be developed for sizing drops in liquid-liquid dispersions. Fiber-optical probe techniques are very fast and promising to be used online.

**Figure 7.**
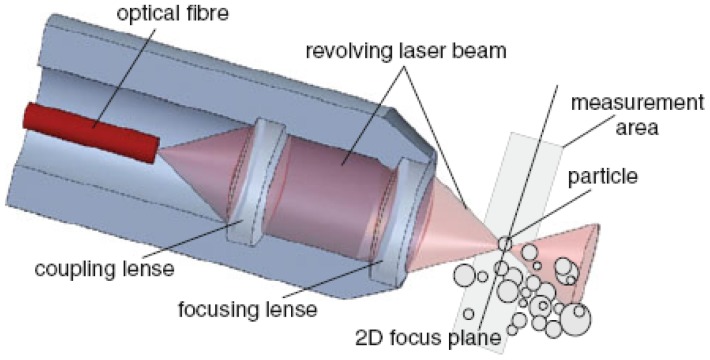
Droplet measurement set-up of the 2D-ORM sensor with 3D working principle [99]. The 2D ORM technique utilizes various laser backscattering technique with an ECA 010 sensor. By ORM technique, an intensive laser light beam is used to obtain the arc chord lengths of emulsion droplets in close vicinity of an optical window at the end of the sensor tube.

**Figure 8.**
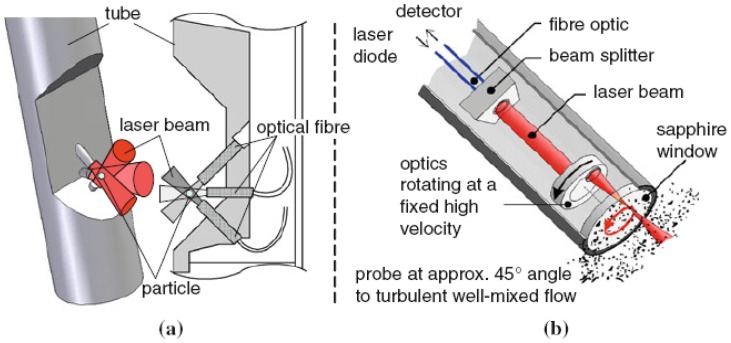
(**a**) Design of the fiber optical FBR sensor: 3D drawing of the tip of the probe (*left*) and the cross-section of the probe (*right*); (**b**) A FBRM probe using light back scattering effects for drop sizing [99].

Various fiber-optical probe techniques based on laser back scattering had been utilized to size droplets in liquid-liquid dispersions for both pipe flow [86,87] and agitated tanks [86,88,89]. Simmons *et al.* [90] tested two optical laser-based drop size measurement techniques (an offline diffraction technique and an online back scattering technique) with glass beads of known size. Both techniques operated satisfactorily only at specific concentration ranges. More authors devoted to the application of the focused beam reflectance measurement (FBRM) probe since it was well-suited for high holdup of the dispersed phase (up to 50% volume fraction). However, the main drawback of the FBRM was found that it did not actually measure the DSD but the chord length distribution (CLD). One should therefore convert the measured CLD to its corresponding DSD. Methods were discussed to transform the measured chord length distribution into a size (diameter) distribution [91–94]. Another study had shown that the FBRM tended to undersize the droplets in emulsion [95]. Greaves *et al.* [95] applied it to emulsions and ice/clathrate hydrate formation processes, and found that certain inaccuracies existed in the chord length distributions. Particularly, the FBRM was found to undersize the droplets in an emulsion and was unable to measure full agglomerate sizes. Also, Boxall *et al.* [96] found that the droplet size was dramatically undersized by the FBRM probe, even taking into account that it measured chord lengths rather than actual sizes. Other laser back scattering techniques are still in test and evaluation. Cull *et al.* [97] and Lovick *et al.* [98] employed a 3D optical reflectance measurement (ORM) technique, which was similar in operation to the FBRM and the 2D optical reflectance measurement (ORM) in a liquid-liquid biocatalytic reactor. Recently, the principles and experimental comparison of three online measurement techniques (the 2D-ORM sensor, the fiber optical FBR sensor and the FBRM probe, shown in Figures 7 and 8) based on laser back scattering fiber-optical techniques for drop size distributions in liquid-liquid dispersions were given and discussed by Maaß *et al.* [99]. It was clearly shown that none provided exact results for the tested toluene/water system. A different measurement principle has to be used for online measurements of drop size distributions than laser back scattering.

**Figure 9.**
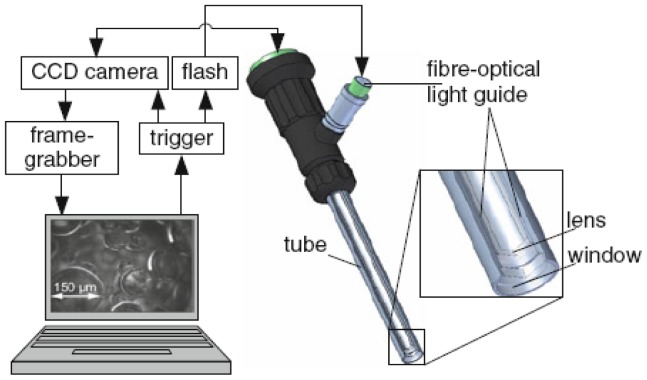
3D drawing of the endoscope probe and general set-up [99]. A strobe flash is guided by a fiber optic cable surrounding the endoscope to ensure sharp pictures even in vicinity of the stirrer.

**Figure 10.**
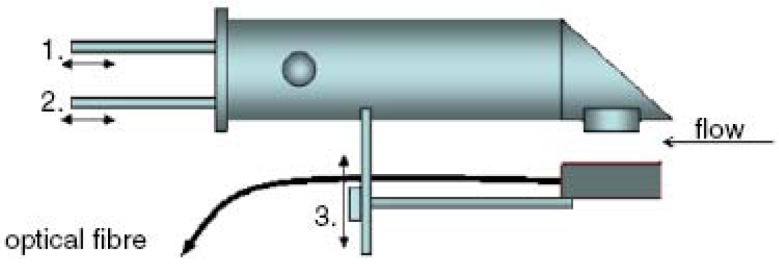
Sketch of the submersible imaging system [102]. 1, 2 and 3 in the figure are three rods to easily adjust the camera position, objective magnification and light diffuser position. The diode laser light is guided through an optical fibre to a back-light diffuser. The imaging system is controlled with a laptop computer and a programmable timing unit.

Fortunately, various vision probe methods have been developed. The vision probe method is the most reliable one since it works with direct image recognition. It gave accurate values for the drop sizes in the analyzed system, and was always used as the “standard” with other probes for comparison [86–88,95,99–101]. Thereinto, the vision probe used by Maaß *et al.* [99] can be regarded as the fiber optics coupled with the digital imaging technique though the fiber optical cables were fixed surrounding the endoscope only to guide the strobe flash for enough sharp pictures as shown in Figure 9. Furthermore, the submersible imaging system including a laptop computer, a CCD camera and a pulsed optical fiber coupled diode laser developed by Honkanen *et al.* may be able to provide a potential application in such area as shown in Figure 10 [102]. The camera is placed on a slide inside a 400 mm long, water proof cylinder whose diameter is 90 mm. The head of the cylinder is skewed to reduce hydrodynamic drag. The coated, inert viewing window is located on the side of the cylinder and there is a surface mirror at 45° at the head of the cylinder. The camera position, objective magnification and light diffuser position can easily be adjusted with three rods. In dense suspensions, the optical path length of the system can be reduced by moving the light diffuser closer to the viewing window. The field of view of the camera is 10–40 mm in width, depending on the application.

### 3.4. Measurements in Gas-Liquid-Solid Systems

In various industrial processes such as petrochemical, biochemical and environmental ones, the complex gas-liquid-solid systems in bubble columns [103], slurry columns [104–107], fluidized beds [108,109], airlift reactors [110], flotation columns and stirred vessels [111,112] are being applied more and more. For a reliable design of such three-phase reactors, it is important to know the complex hydrodynamics (*i.e.*, phase-phase interactions) associated with the demands of simultaneous gas dispersion and solids suspension.

The fiber-optical probes were primarily developed in the frame of gas-liquid and liquid-liquid flows [113]. The presence of solid phase can influence the gas-liquid mixture in different ways such as bubble rise and formation, radial [114] and axial profiles, mixing and dispersion, gas holdup and flow regimes [115,116], and mass transfer [117–119]. So the measurements in such complex flows must be performed with caution especially in dense gas-liquid-solid flows.

**Figure 11.**
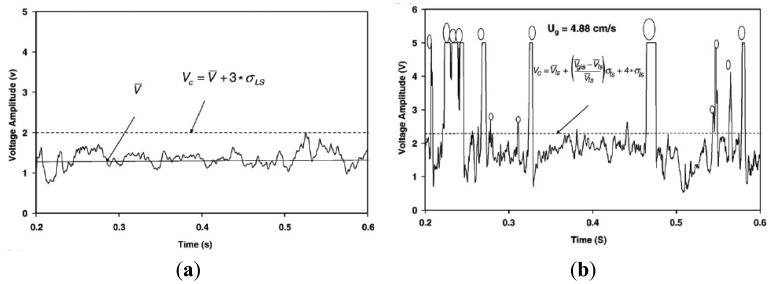
The typical voltage output signal produced by an optical fiber probe for (**a**) liquid-solid; and (**b**) gas-liquid-solid system in a GLSCFB riser for glass beads [127]. In a liquid-solid system, the critical voltage range (*V_c_*), the signal *V* above which indicates a detected particle, is the average voltage (*V̄*) plus 3 times the standard deviation of the liquid-solid system (*σ***_LS_**). In a gas-liquid-solid system, the criterion for gas bubble detection is similarly determined as $V_{c}={\bar{V}}_{\text{LS}}+\left(\frac{{\bar{V}}_{\text{GLS}}-{\bar{V}}_{\text{LS}}}{{\bar{V}}_{\text{LS}}}\right)\sigma_{\text{LS}}+4\sigma_{\text{LS}}$, in which the average and standard deviation of LS and GLS systems are used.

In gas-liquid-solid systems, bubble dynamics plays a key role in dictating the transport phenomena and ultimately affects the overall rates of chemical reaction. Lee *et al.* [120] and Lee and de Lasa [121] measured the local gas volume fraction and bubble frequency in a three-phase fluidized bed using the U shape optical fiber probe. Yu and Kim [122] applied the U shape optical fiber probe to study the radial distributions of bubble size, bubble rise velocity and bubble volume fraction in three-phase fluidized beds. Frijlink [123] developed a four-point probe to improve the detection of the direction of the movement and the shape of the bubble. Chabot and de Lasa [124] measured the axial and radial distributions of bubble chord length, bubble rise velocity and gas volume fraction in a bubble column at high temperature by using the refractive optical probe. Shoukri *et al.* [125] measured the gas volume fraction, bubble size, bubble rise velocity, bubble frequency and interfacial area in a large scale bubble column using a dual optical probe. Wang *et al.* [126] used a fiber-optical probe consisting of two parallel optical fibers to investigate the local gas holdup, bubble size distribution, and bubble rise velocity in different radial positions. In a recent study by Razzak *et al.* [127], the fiber-optical probe, namely PV-5, capable of measuring solids concentration in two- or three-phase fluidized beds was extended to measure bubble holdups. As shown in Figure 11, the output voltage in the presence of bubbles was higher due to the reflection of light beams over the bubbles and any signal with voltage larger than the critical voltage range was the indication of a gas bubble. Mena *et al.* [128] measured simultaneously gas phase residence time and gas phase velocity using a mono-fiber optical probe, manufactured at LEGI, typically for the bubble measurement in gas-liquid flows. Besides, Razzak *et al.* [61] coupled the electrical resistance tomography with a fiber-optical probe to measure the gas holdup in three-phase fluidized beds.

**Figure 12.**
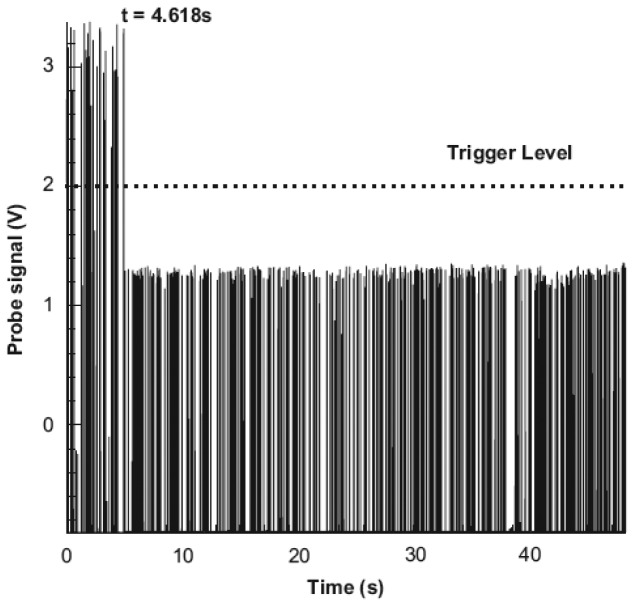
Probe signal during particle and bubble piercing (long time acquisition) [128]. As the probe tip was “contaminated” by small particles (*t* ≤ 4.618 s), the light reflection decreased, which led to underestimated measurement.

Seemingly, reliable fiber-optical probe techniques had been built up for a gas-liquid-solid fluidized bed or bubble column, and the bubble and solid particle could be easily distinguished by an analysis of signal data. However, negative interference of particles on probe function should have been focused on but were less done. Actually, when a solid particle passes by the fiber-optical probe, it may also be pierced by the probe or adhere to the surface of the probe based on its properties such as, wettability, *etc.* Then the probe tip is contaminated with small solids adhered to the probe. This status may often occur during the experimental measurements in multiphase reactors under industrial relevant operating conditions. Mena *et al.* [128] found a calcium alginate particle was pierced by the optical probe when it passed by the probe. As the tip was “contaminated” by small particles the light reflection increased, leading to high amplitude signal shown in Figure 12 (*t* ≤ 4.618 s). It is worse that the tip might be parceled entirely by the solid particles. For example, in a crystallization reactor, the probe tip was taken as the crystal core and the crystal grew up gradually on the surface of the probe tip. So, more investigations are still needed to develop and test reliable measuring techniques for the measurements under industrial relevant operating conditions.

For the reliable design of a three-phase stirred tank reactor, it is important to know the complex hydrodynamic properties (*i.e.*, phase-phase interactions) associated with the demands of simultaneous gas dispersion and solids suspension with a single impeller or multiple one [129]. In such a reactor, flows are more complex and chaotic (dense dispersed-phase holdup and liquid flow rates, large bubbles and vortices, interactions between gas and solid, *etc.*). Up to now, there has no successful study on the bubble measurement in a three-phase stirred tank using a fiber-optical probe technique though a lot have been conducted in a gas-liquid one such as Wang *et al.* [130]. It is an available method to judge the solid suspension based on the information about the solids concentration profiles (axial or radial) or the solids concentration distribution existing in the stirred vessel. Various methods had been reported in the literature for the measurement of solids concentration in a solid-liquid stirred vessel. Besides the expensive visual methods, the others including sampling technique [131–133], conductivity method [134], optical method [135] *etc.* also have their respective shortcomings. None of mature fiber-optical methods can be used reliably for reference.

**Figure 13.**
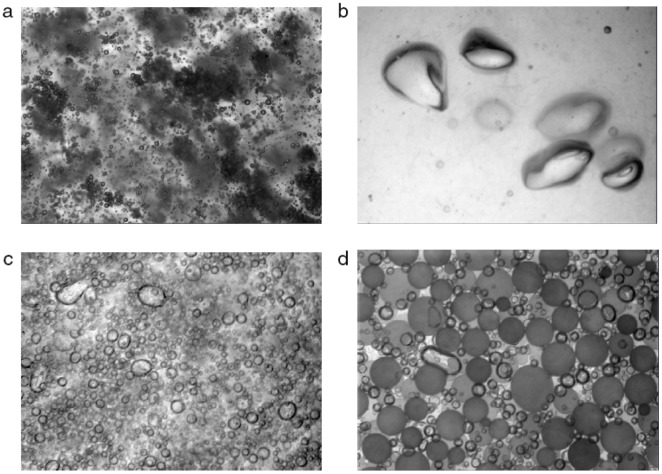
(**a**) Micro-bubbles and flocculate waste particles in an industrial DAF process; (**b**) Bubbles and fibers in a white water de-aeration channel flow; (**c**) Bubbles in the inlet flow of a deinking flotation process; (**d**) Plastic beads and gas bubbles in a plastic bead production reactor [102].

In future, the vision probe methods may be the most promising, such as the ones developed by Maaß *et al.* [99] and Honkanen *et al.* [102] in a gas-liquid-solid reactor. Especially, the vision probe developed by Honkanen *et al.* [102] aimed at the investigation of multiphase flows in various industrial applications. The submersible imaging system in Figure 10, had been utilized in experiments for four three-phase industrial applications: (1) waste water purification in a dissolved air flotation (DAF) tank; (2) bubbly wood fiber suspension in a white water de-aeration channel; (3) recycled pulp suspension in a deinking flotation cell; (4) a plastic bead production reactor, as shown in Figure 13. Mobile phone technology has encouraged the production of small camera sensors with several megapixels resolution and also opens a new world for the development of the vision fiber-optical probe.

## 4. Conclusions and Perspectives

This work presents a brief introduction on the fiber-optical sensor basics and an overview focusing on the applications to the measurements in multiphase reactors. Relatively, the fiber-optical probe techniques in two-phase reactors are better developed but more efforts are still needed to make them perfect. In gas-liquid reactors, the techniques on the measurement in multi-dimensional, complex and chaotic, and organic flows have been explored. Though the results obtained are still unsatisfactory, they are valuable attempts for the measurements under relevant industrial operating conditions and also point out the developing direction in the future. The fiber-optical probe techniques in gas-solid and liquid-solid reactors are the most mature. Measurements of transient or evolving drop characteristics demand the online techniques. Up to now, none of the fiber-optical probe techniques based on laser back scattering provided exact results for the tested system. A different measurement principle has to be developed and used for online measurements of drop size distributions than laser back scattering or the vision probe is directly adopted. There are many studies on the measurements of gas holdup using fiber-optical probes in three-phase fluidized beds. However, the interactions between solids and probe tips should have been focused on but were less done because glass beads *etc.* were always used as the solid phase. The vision probe methods may be the most promising for simultaneous gas dispersion and solids suspension measurement.

Based on the status quo of applications in multiphase reactors, the fiber-optical probes techniques should be developed further in the following directions:

1. Online measuring techniques under nearly industrial operating conditions
  The online techniques can obtain the transient characteristics. The measurement under nearly industrial operating conditions is of significance to design and scale up of multiphase reactors. The importance of online measuring techniques under nearly industrial operating conditions has evoked more and more interests.
2. Corresponding signal data processing techniques
  A mass of signal data are encounterred with an online fiber-optical probe technique. So the quick processing techniques are very essential. Under nearly industrial operating conditions, the flows in multiphase reactors may be complex and chaotic. The signal processing techniques and the assumptions such as exclusively vertical bubble motion, isotropy of turbulence and regular bubble shapes (spherical or ellipsoidal) may be not exact and unsuitable. New signal data processing techniques are needed.
3. Coupling with other measuring techniques.
  The applications of the fiber-optical probe method in various multiphase reactors have their own limitations. For example, the bubble velocity near the wall is so lower that the bubble can not be pierced by the probe tip. Thus, this bubble will escape from the detection of the fiber-optical probe. Coupled with other measuring techniques, the fiber-optical probe method will be more robust and can eliminate some limitations. Just as Razzak *et al.* [61], the ERT can be used to detect the bubble characteristics near the wall and the bubbles in other zones are measured by the fiber-optical probe in gas-liquid systems. In practice, fiber-optical probe techniques can be coupled with all optical techniques, thus increasing their versatility. The vision probe methods may be the most promising and able to provide a potential application in multiphase, especially liquid-liquid and gas-liquid-solid, reactors.

## Figures and Tables

**Table 1.** Fiber-optical sensor classifications under three categories.

**Category**	**Class**	**Trait**
sensing location	point sensors	with a sensitized tip in the measurand field to measure along the length of the fiber itself “in between” point and distributed sensors
	distributed sensors	
	quasi-distributed sensors	
operating principle	intensity sensors	
	phase sensors	
	frequency sensors	
	polarization sensors	
application	physical sensors	for temperature, stress, velocity, *etc.*
	chemical sensors	for pH, gas analysis, spectroscopic studies, *etc.*
	bio-medical sensors	for blood flow, glucose content, *etc.*

## Abbreviations

BSD: bubble size distribution
CCD: charge-coupled device
CFD: computational fluid dynamic
CLD: chord length distribution
DAF: dissolved air flotation
DSD: drop size distribution
ECT: electrical capacitance tomography
EMI: electromagnetic interference immunity
ERT: electrical resistance tomography
FBR: forward backward ratio
FBRM: focused beam reflectance measurement
GLSCFB: gas-liquid-solid circulating fluidized bed
IAC: interfacial area concentration
LED: light emitting diode
LSCFB: liquid-solid circulating fluidized bed
ORM: optical reflectance measurement
PBE: population balance equation
UV-VIS: ultraviolet-visible
